# Assembly Patterns of the Rhizosphere Microbiome Along the Longitudinal Root Axis of Maize (*Zea mays* L.)

**DOI:** 10.3389/fmicb.2021.614501

**Published:** 2021-02-12

**Authors:** Lioba Rüger, Kai Feng, Kenneth Dumack, Jule Freudenthal, Yan Chen, Ruibo Sun, Monica Wilson, Peng Yu, Bo Sun, Ye Deng, Frank Hochholdinger, Doris Vetterlein, Michael Bonkowski

**Affiliations:** ^1^Terrestrial Ecology, Institute of Zoology, University of Cologne, Cologne, Germany; ^2^CAS Key Laboratory for Environmental Biotechnology, Research Center for Eco-Environmental Sciences, Chinese Academy of Sciences, Beijing, China; ^3^College of Resources and Environment, University of Chinese Academy of Sciences, Beijing, China; ^4^Institute of Soil Science, Chinese Academy of Sciences, Nanjing, China; ^5^Microbial Ecology Lab, Center for Agricultural Resources Research, Institute of Genetics and Developmental Biology, Chinese Academy of Sciences, Shijiazhuang, China; ^6^Crop Functional Genomics, Institute of Crop Science and Resource Conservation (INRES), University of Bonn, Bonn, Germany; ^7^Department of Soil System Science, Helmholtz Centre for Environmental Research – UFZ, Halle, Germany; ^8^Soil Science, Martin-Luther-University Halle-Wittenberg, Halle, Germany

**Keywords:** archaea, bacteria, Cercozoa, microbial assembly, plant microbiome, protists, rhizosphere

## Abstract

It is by now well proven that different plant species within their specific root systems select for distinct subsets of microbiota from bulk soil – their individual rhizosphere microbiomes. In maize, root growth advances several centimeters each day, with the locations, quality and quantity of rhizodeposition changing. We investigated the assembly of communities of prokaryotes (archaea and bacteria) and their protistan predators (Cercozoa, Rhizaria) along the longitudinal root axis of maize (*Zea mays* L.). We grew maize plants in an agricultural loamy soil and sampled rhizosphere soil at distinct locations along maize roots. We applied high-throughput sequencing, followed by diversity and network analyses in order to track changes in relative abundances, diversity and co-occurrence of rhizosphere microbiota along the root axis. Apart from a reduction of operational taxonomic unit (OTU) richness and a strong shift in community composition between bulk soil and root tips, patterns of microbial community assembly along maize-roots were more complex than expected. High variation in beta diversity at root tips and the root hair zone indicated substantial randomness of community assembly. Root hair zone communities were characterized by massive co-occurrence of microbial taxa, likely fueled by abundant resource supply from rhizodeposition. Further up the root where lateral roots emerged processes of community assembly appeared to be more deterministic (e.g., through competition and predation). This shift toward significance of deterministic processes was revealed by low variability of beta diversity, changes in network topology, and the appearance of regular phylogenetic co-occurrence patterns in bipartite networks between prokaryotes and their potential protistan predators. Such patterns were strongest in regions with fully developed laterals, suggesting that a consistent rhizosphere microbiome finally assembled. For the targeted improvement of microbiome function, such knowledge on the processes of microbiome assembly on roots and its temporal and spatial variability is crucially important.

## Introduction

The predictable assembly of specific subsets of the soil microbiota in the rhizosphere of plants has led to the characterization of plant species-specific “microbiomes” (Hirsch and Mauchline, 2012; Lundberg et al., 2012; Peiffer et al., 2013; Berg et al., 2014). As the microbiome concept implies a rather static outcome of microbial assembly processes, it raises the question as to where and how the dynamic transition of a microbial bulk soil community into a plant species-specific rhizosphere microbiome is taking place.

Differences in resource supply (bottom–up processes) are thought to be the main driver of microbiome assembly (Edwards et al., 2015; van der Heijden and Schlaeppi, 2015). The vast majority of microorganisms in bulk soil rest in an inactive dormant state of starvation, because their activity is severely limited by the availability of energy from readily accessible carbon molecules (Blagodatskaya and Kuzyakov, 2013). This carbon limitation is temporarily offset by pulses of exudates released by the growing root that triggers the bulk soil bacteria into activity (Boddy et al., 2007). Several studies have demonstrated that it takes between 6 and 10 h until bacteria have switched their metabolism from dormancy to active growth (Anderson and Domsch, 1985; Blagodatskaya et al., 2009; Bonkowski and Clarholm, 2012). However, root exudation does not stimulate rhizobacteria uniformly, rather, rhizodeposition selects for certain fast-growing, copiotrophic bacterial taxa (Maloney et al., 1997; Zelenev et al., 2005; Fierer et al., 2007), leading to reduced taxonomic diversity in the rhizosphere compared to bulk soil (Bulgarelli et al., 2012; Shi et al., 2015; Fan et al., 2017). It is thought that differences in the amount and composition of rhizodeposits further select for the adapted plant species- and genotype-specific bacterial microbiomes (Bais et al., 2006; Hartmann et al., 2009; Bulgarelli et al., 2012).

The dynamic nature of root systems contrasts with the rather static perception of microbiomes. Plant roots, and the carbon sources they provide, are far from uniform. Rhizodeposition is locally restricted to specific root regions, which undergo continuous transformation (Watt et al., 2006). At their root tips, plants slough off root cap cells (Hawes, 1991; Iijima et al., 2000) and secrete mucilage (Osborn et al., 1999; Knee et al., 2001). Further up the root, small molecular weight exudates are passively released mainly via root hair cells and leak out where laterals emerge (Jaeger et al., 1999; Farrar et al., 2003; Walker et al., 2003; Shaw et al., 2006). Individual primary and seminal roots of maize typically grow around 2–3 cm day^–1^ but up to 7 cm day^–1^ (Watt et al., 2006; de Moraes et al., 2019) therefore suggesting a continuous process of community re-assembly and sustained microbial invasions from bulk soil along the advancing root.

Community assembly theory assumes randomness through priority effects, in which earlier arriving species gain a competitive advantage over subsequent niche inhabitants through exploitation (niche preemption) or manipulation of rhizodeposition (niche modification) along the growing root axis (Fukami, 2015). Randomness through niche preemption can be significantly reduced if competitiveness between taxa differs (Tovi et al., 2019). For example, Lugtenberg et al. (2001) identified the rapid colonization of root tips as a key trait of successful “rhizosphere competent” bacteria, suggesting that microbiome assembly will be shaped already at the root apex. In correspondence Dupuy and Silk (2016), concluded from their model that attachment to root tips was a key bacterial trait that gave bacteria greatest access to exudate carbon and significantly increased their proliferation along the root. In contrast, priority effects through niche modification as shown for the gut microbiome (Sprockett et al., 2018), appear also common in the rhizosphere, where microorganisms influence the composition of root exudates (Naher et al., 2008), change root immune responses and trigger the release of quorum sensing mimics by plant roots (Mathesius et al., 2003; Bauer and Mathesius, 2004; Barnard et al., 2007), or even manipulate the whole root architecture (Hartmann and Schikora, 2012). The early colonizers may modify the environment for later-arriving species to such an extent that assembly history may become the dominant driver of community assembly (Fukami, 2015).

As root growth progresses, the initially random community assembly at the root tip is expected to be replaced at some point by increasingly deterministic processes that lead to the typical plant-associated microbiomes. Deterministic assembly of microbial communities can arise through two mechanisms. The first is competition for resources, a mechanism that is likely to increase when rhizodeposits subside (Nemergut et al., 2013). The second is through selection, by both the plant immune system at the rhizoplane (van der Heijden and Schlaeppi, 2015) and its surrounding rhizosphere where predation of bacterivores (top–down processes) leads to deterministic patterns in reproduction and mortality rates of individual bacterial taxa (Matz and Kjelleberg, 2005; Jousset, 2012). Especially the omnipresent predation of bacterivore protists exerts substantial selection pressure on rhizosphere bacteria (Jousset et al., 2008, 2010; Rosenberg et al., 2009; Henkes et al., 2018), thereby altering functional traits such as increasing grazing resistance and biocontrol compounds (Jousset et al., 2006, 2010; Jousset and Bonkowski, 2010; Jousset, 2012; Flues et al., 2017). However, bacterial investment in defenses carries a significant growth-defense tradeoff (Riley and Wertz, 2002; Denison et al., 2003; Corno and Jürgens, 2008; Jousset et al., 2009) thus redirecting competitive outcomes in bacterial communities under selection pressure of protistan bacterivores (Matz and Jurgens, 2003; Jousset et al., 2008, 2009).

To gain insights into the self-organization of the rhizosphere microbiome, we investigated the assembly of rhizosphere specific microbial communities of prokaryotes and their protistan predators along the longitudinal root axis of maize plants at clearly defined root regions. We investigated the early assembly at root tips to communities of subsequently older root regions until first order lateral roots dominated root architecture. We hypothesized an immediate reduction of alpha diversity of prokaryotes in rhizosphere as compared to bulk soil due to a competitive advantage of the fast-growing, copiotrophic taxa from rhizodeposition. Differences in beta diversity, however, should be maximized on root tips, due to an increased likelihood of random priority effects in the early stages of community assembly. Further on, we expected that root regions with highest resource availability (e.g., root tips and the root hair region) would favor many taxa indiscriminately, becoming visible by increased evenness and in networks by high positive co-occurrence of taxa. Finally, we expected the strongest selective forces when rhizodeposits subside, both by microbial competition and predation, which eventually leads to the assembly of the microbiome. Since the “microbiome” is predicated on deterministic processes, it would be characterized by the strongest shift in community structure (beta diversity) compared to bulk soil, together with a strongly reduced variation of beta diversity (i.e., high determinism). At the same time, co-occurrence networks would show a more structured topology, on the one hand due to competitive exclusion and on the other hand by taxa which coevolved during the assembly process and can mutually coexist in the rhizosphere. The existence of top-down processes by protists through selective grazing should be revealed in potential trophic networks between prokaryotes and protists by clear phylogenetic patterns of both negative co-occurrences (i.e., less defended taxa) as well as positive co-occurrences (i.e., well defended taxa benefiting from protists feeding on their competitors).

## Materials and Methods

### Experimental Set Up

*Zea mays* seeds (inbred line B73) were surface sterilized with 10% H_2_O_2_ for 10 min and germinated on wet filter paper under sterile conditions at 18°C in the dark. After 3 days, 40 seedlings of similar length were selected and separately planted into cylindrical perspex microcosms (250 mm height, 70 mm inner diameter) filled with 885 cm^3^ of 1 mm sieved sandy loam soil at a bulk density of 1.46 ± 0.1 g dry wt cm^–3^, corresponding to 1300 ± 80 g dry wt microcosm^–1^. The bottom of each microcosm was closed with a nylon gauze (30 μm mesh size) for watering. Soil water content was kept constant at 22%_VWC_ by daily weighing and replacement of water. Tubes were wrapped in aluminum foil to protect soil and roots from light. Plants were grown in a climate chamber with a day–night regime of 12/12 h (350M PAR) at 24°C/18°C and 65% humidity.

### Sampling

**FIGURE 1:**
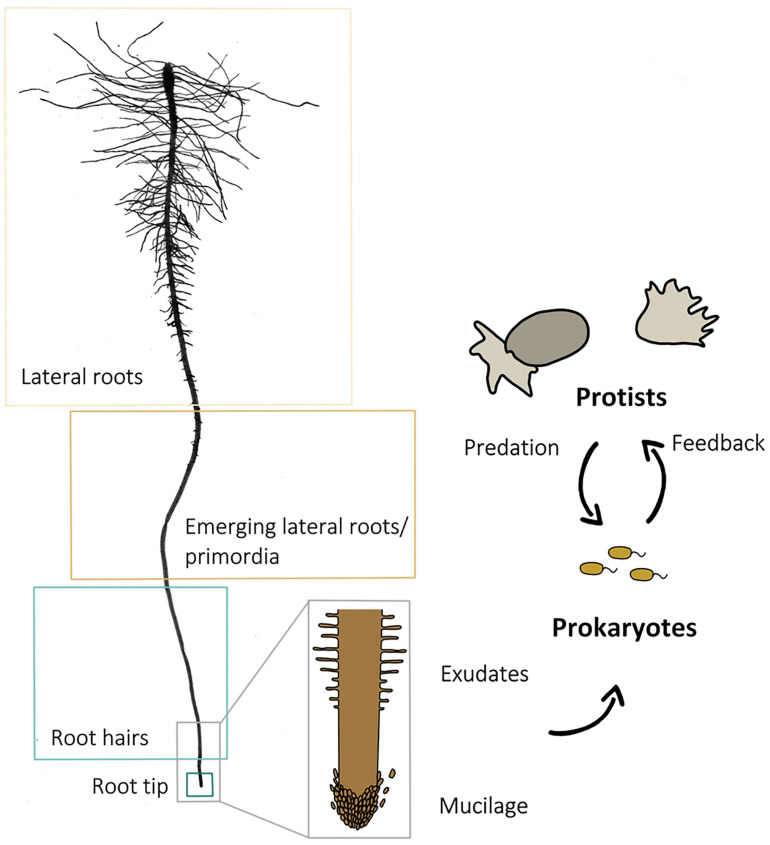
Scan of a washed primary root of an experimental plant and scheme of a root with root cap cells at the tip and root hairs above. The arrows indicate the direction of the influence different components (root, prokaryotes, and protists) exert. The four sampled root regions (lateral roots, lateral root primordia, root hair zone, and root tip) are framed for identification.

Microcosms were sampled 9 days after planting. Using sterile forceps and scissors, 1 cm root samples with adhering soil from (i) the root tip, (ii) the root hair zones, (iii) the region where the first lateral root primordia emerged as earliest lateral roots, and (iv) from a subsequent region with fully developed lateral roots (Figure 1) were transferred into sterile 15 ml centrifuge tubes. Samples of three different roots of the same plant were pooled to one biological replicate. As a control, a bulk soil sample was taken from each microcosm, consisting of five randomly chosen and pooled soil samples which were not in the direct vicinity of a root. Each root region and bulk soil sampling was replicated 40 times. Soil was washed off from roots by vortexing in a 0.3%NaCl solution. After 30 min of centrifugation at 5000 × *g*, the supernatant was discarded, and the pellet was used for DNA extraction. DNA-extraction and purification were done using the FastDNA^®^ SPIN Kit for soil and the GENECLEAN^®^ SPIN Kit (MP Biomedicals, Santa Ana, CA, United States), following the manufacturer’s instructions.

### Cercozoa

#### Amplicon-Sequencing

A two-step PCR yielding a c. 350 bp long fragment of the V4 region of the SSU/18S gene was conducted using primers (Fiore-Donno et al., 2020) targeting amplification of Cercozoa. In a first PCR a mixture of identical amounts of the forward primers S615F_Cerco (5′-GTTAAAAAGCTCGTAGTTG-3′) and S615F_Phyt (5′-GTTAAAARGCTCGTAGTCG-3′) together with the reverse primer S963R_Phyt (5′-CAACTTTCGT TCTTGATTAAA-3′) were used. In a second, semi-nested PCR, samples were indexed by using the forward primer S615F_Cer (5′GTTAAAARGCTCGTAGTYG-3′) and the reverse primer S947R_Cer (5′-AAGARGAYATCCTTGGTG-3′), both tagged with barcodes as described by Fiore-Donno et al. (2020).

A 1 μl of purified DNA from soil samples served as the template for the first PCR, resulting in amplicons of which 1 μl was used for the second semi-nested PCR. Both PCR-rounds were conducted with reagents in the following final concentrations: Dream Taq polymerase (Thermo Fisher Scientific, Dreieich, Germany) 0.01 units, Thermo Scientific Dream Taq Green Buffer, dNTPs 0.2 mM and primers 1 μM. All PCRs were conducted in duplicates to reduce the possible artificial dominance of few amplicons by PCR competition and then pooled. The thermal program for the reaction started with a 2 min denaturation step at 95°C, followed by 95°C for 2 min, (95°C for 30 s, 50°C for 30 s, 72°C for 30 s) repeated 24 times and a final elongation step at 72°C for 5 min. After checking for successful amplification and potential contamination by gel electrophoresis, 24 μl of the PCR-product of each sample were purified and normalized using SequalPrep Normalization Plate Kit (Invitrogen GmbH, Karlsruhe, Germany) to obtain a concentration of 1–2 ng/μl per sample. Purified amplicons were pooled, concentrated and sequenced on an Illumina MiSeq platform (Illumina Inc., San Diego, CA, United States) at the Cologne Center for Genomics (Cologne, Germany). With the MiSeq v3 Reagent kit, 2 × 300 cycles were performed, producing 300 bp long paired-end reads.

#### Sequence Processing

Reads were processed using the customized MOTHUR pipeline v.39.5 (Schloss et al., 2009).

Paired-end reads were merged, not allowing any mismatches in primer or barcode sequences, maximum two mismatches and one ambiguity in the target sequence. Assembled sequences with an overlap <200 bp were removed. Merged contigs were demultiplexed and primer and tag sequences were trimmed. Remaining reads were clustered into operational taxonomic units (OTUs) using VSEARCH (Rognes et al., 2016) according to the abundance-based greedy algorithm (agc) with a similarity threshold of 97%. Clusters represented by less than 500 reads were removed as likely to represent amplification or sequencing noise (Fiore-Donno et al., 2018). OTUs were assigned to taxa using BLAST+ (Camacho et al., 2009) with an e-value of 1^*e*–50^ and the PR2 database (Guillou et al., 2013), keeping only the best hit. Non-target sequences (12 of 723 OTUs were excluded. Sequences were aligned with the provided template (Fiore-Donno et al., 2018), allowing gaps of maximum five nucleotides and cleaned from chimeras using UCHIME (Edgar et al., 2011), resulting in 513 OTUs. The sampling depth reached up 17,062 sequences per sample.

### Bacteria/Archaea

#### Amplicon-Sequencing

The forward primer 515F (5′-GTGCCAGCMGCCGCGGTAA-3′) (Caporaso et al., 2011) and the reverse primer 806R (5′-GGACTACNVGGGTWTCTAAT-3′) (Apprill et al., 2015), targeting a c. 390 bp long fragment of the V4 region of the SSU/16S genes of bacteria and archaea were used for amplification. The PCR was set up with 2 μl of template DNA, 12.5 μl of TaKaRa Premix Taq^TM^ (TaKaRa Bio Group) and 10 μM of forward and reverse primer each. The thermal program for the reaction started with a 5 min denaturation step at 95°C, followed by (94°C for 30 s, 56°C for 30 s, 72°C for 40 s) repeated 30 times and a final elongation step at 72°C for 5 min. The sequencing was done on the Illumina HiSeq platform (Illumina Inc., San Diego, CA, United States) by Magigene Technology Co., Ltd. (Guangzhou, China). With a HiSeq v2 Reagent kit, 2 × 250 cycles were performed, producing 250 bp long paired-end reads.

#### Sequence Processing

Quality trimming and adapter clipping of the raw reads was performed using Trimmomatic (Bolger et al., 2014). Paired-end reads were merged using fastq-join (Aronesty, 2011) with a minimum overlap of 10 bp and maximum 10% difference within the overlapping region. Reads containing errors in primer sequences were discarded, and primer sequences were removed using cutadapt (Martin, 2011). Chimeric reads were filtered and removed with UCHIME (Edgar et al., 2011). Subsequently VSEARCH (Rognes et al., 2016) was used to cluster reads into OTUs with a similarity threshold of 97%. To assign taxa to OTUs the RDP Classifier was used with the Silva database (version 132) as reference. OTUs which were not assigned to bacteria or archaea as well as those represented by less than 100 reads were removed to get rid of erroneous sequences. Read counts were subsampled to the minimum number of reads (50,438) in a sample.

### Statistical Analysis

Statistical analyses and data visualization were performed in R version 3.5.1 (R Core Team, 2018). With our sequencing depth we reached saturation in taxon sampling (Supplementary Figure 1). For downstream analysis, the total number of reads was transformed into relative abundances. Species richness, evenness and alpha diversity were compared using Welch’s one-way ANOVA, followed by a Games-Howell non-parametric *post hoc* test. After the removal of one outlier from the prokaryote and two from the cercozoan dataset permutational multivariate analysis of variance (PERMANOVA) using Bray–Curtis dissimilarity, permuted 999 times, was employed to test for differences in prokaryote and cercozoan community structure across samples from different root regions.

### Network Analysis

Co-occurrence networks incorporating communities containing bacteria, archaea, and Cercozoa were based on single OTUs and generated to assess co-occurrence or potential interactions between species. To assess the complexity and specificity of community structures along roots, network analyses were conducted for communities at the four different sampled root regions and from bulk soil. For network construction and analysis of topological features the molecular ecological network analysis pipeline (MENAP)^1^ (Zhou et al., 2011; Deng et al., 2012) was used. From OTUs which occurred in more than 75% of the samples within each group a Spearman rank correlation matrix without log-transformation was calculated. Based on random matrix theory a threshold of 0.76 was determined in MENAP. When the correlation coefficients were higher than 0.76, interactions were considered as significant positive, when they were lower than −0.76 as significant negative. As implemented in the MENAP topological features as total number of nodes (OTUs), total number of links, *R*^2^ of power law, average connectivity (or average degree) which measures the complexity of a network, average clustering coefficient, average path distance and modularity were calculated. Based on detected modules among-module connectivity and inter-module connectivity were calculated, and nodes were assigned to one of four possible network roles: network hubs, module hubs, connectors or periphers.

In order to characterize the potential impact of protists on prokaryote community assembly along roots, inter-domain associations between Cercozoa and prokaryotes were extracted from full networks. From these, bipartite networks were generated with nodes displaying cercozoan OTUs, grouped at family and prokaryote OTUs, grouped at phylum level. The network topological features of these bipartite networks were calculated using the Interdomain Ecological Network Analysis Pipeline (IDENAP)^2^, including connectance which is the proportion of possible links that are established, links per OTU, number of compartments, and modularity for the cercozoan community. The network graphs were visualized in Cytoscape 3.7.2 (Shannon et al., 2003).

## Results

### Community Structure

**FIGURE 2:**
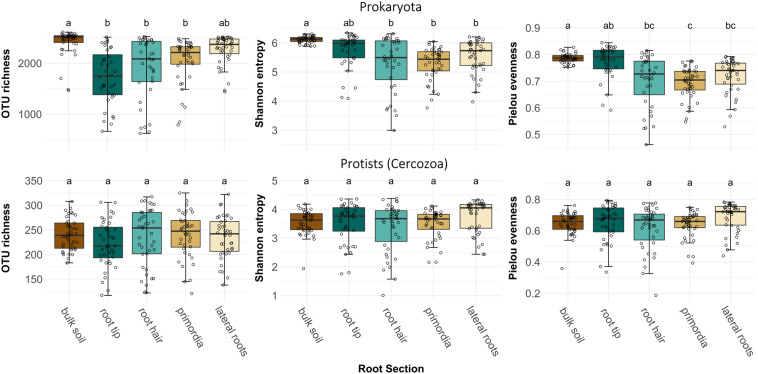
Operational taxonomic unit richness, alpha diversity (Shannon index) and evenness (Pielou’s index) of prokaryote and protist (cercozoan) communities in bulk soil and at four different root regions of maize plants. Different letters indicate differences between means (Welch’s one-way with Games-Howell *post hoc* test). Prokaryote OTU richness: *F*_4,186_ = 14.12, *p* < 0.001, prokaryote Shannon diversity: *F*_4,186_ = 12.88, *p* < 0.001, prokaryote Pielou’s evenness: *F*_4,186_ = 16.71, *p* < 0.001, protist OTU richness: *F*_4,193_ = 1.09, *p* > 0.05, protist Shannon diversity: *F*_4,193_ = 1.99, *p* > 0.05, and protist Pielou’s evenness: *F*_4,193_ = 2.62, *p* = 0.36.

After sequence processing 513 cercozoan and 3355 prokaryotic OTUs were obtained. OTU richness of prokaryotes was highest in bulk soil, but dropped significantly when encountered by a root tip, and then only gradually increased again toward the older root regions (Figure 2, ANOVA, *F*_4,186_ = 14.12, *p* < 0.001). Bulk soil and root tips showed highest prokaryote alpha diversity (ANOVA, *F*_4,186_ = 12.88, *p* < 0.001) and evenness (ANOVA, *F*_4,186_ = 16.71, *p* < 0.001) compared to older root regions (Figure 2). However, OTU richness, alpha diversity and evenness of root-associated prokaryotes showed substantially higher variation than in bulk soil (Supplementary Table 1). In contrast to prokaryotes, protist richness and alpha diversity did not differ between bulk soil and root regions (Figure 2).

**FIGURE 3:**
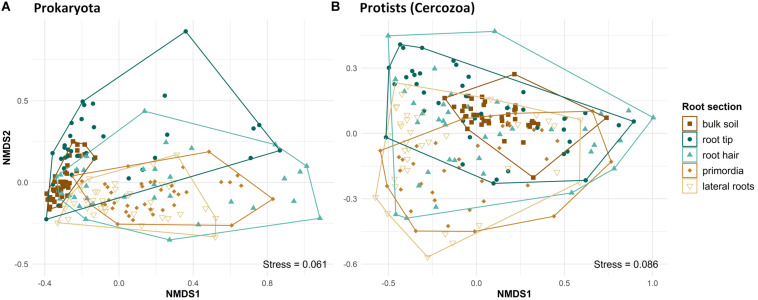
Ordination of NMDS, displaying Bray–Curtis dissimilarities of prokaryote (PERMANOVA: *F*_4,185_ = 12.134, *R*^2^ = 0.218, *p* = 0.001) and protist (cercozoan) (PERMANOVA: *F*_4,193_ = 4.465, *R*^2^ = 0.0847, *p* = 0.001) communities in bulk soil and at four different root regions from microcosms. Symbol colors and shapes in legend separating bulk soil and the respecting root regions.

Beta diversity differed significantly between bulk soil and root regions and among different root regions, in both prokaryotes (PERMANOVA, *F*_4,185_ = 12.13, *R*^2^ = 0.22, *p* < 0.001) and protists (PERMANOVA, *F*_4,193_ = 4.47, *R*^2^ = 0.085, *p* < 0.001). Beta diversity of prokaryote communities was lowest in bulk soil, highest at root tips and the root hair zone, and decreased again toward lateral root primordia and lateral roots, respectively (Figure 3, NMDS plot). Pairwise comparisons revealed differences in community structure of prokaryotes in bulk soil and at root tips compared to all other root regions and between the communities of lateral root primordia compared to lateral roots (Supplementary Table 2). Protist community structure differed significantly between bulk soil and root tips (Supplementary Table 3) and generally reflected the pattern seen in NMDS of prokaryotes although shifts in beta diversity from root tips to increasingly older root regions showed more variation (Figure 3B).

### Network Analysis

**FIGURE 4:**
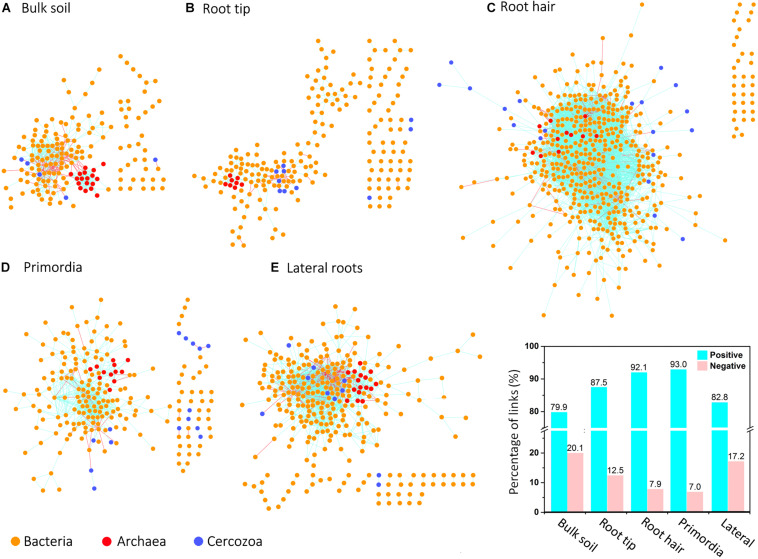
Microbial co-occurrence networks based on correlation analysis of bacteria (yellow points), archaea (red points), and Cercozoa (blue points) in **(A)** bulk soil, **(B)** root tip, **(C)** root hair zone, **(D)** lateral root primordial, and **(E)** lateral roots. Connections show positive correlations in turquoise and negative correlations in pink. The bar plot shows the percentage of positive and negative correlations between Cercozoa, bacteria and archaea in bulk soil and at the different root regions.

Network topologies of bulk soil and the four root regions differed significantly from randomly generated networks (Tables 1, 2). Network connectivity followed a power law (*R*^2^ from 0.954 to 0.994), indicative of a scale-free network structure with a topology of many nodes with few connections and some highly connected nodes within modules (i.e., small values of average path distance, except at root tips; Table 1). The total number of nodes was lowest in bulk soil (172; Table 1) and reached a threefold higher maximum in the root hair zone (511; Table 1). Furthermore, the total number of links was lowest at root tips (279 links; Table 1) but increased 23-fold in the subsequent root hair zone (6396 links, Table 1). Average degree and the average clustering coefficient were lowest and average path distance was highest in root tip networks (Table 1), indicating poorly structured, weakly correlated and widely dispersed networks with few links (Figure 4). In contrast, networks of the root hair zone showed by far the highest average degree, indicating the highest connectivity of OTUs, but the lowest modularity and average path distance (Table 1) suggesting a barely sub-structured and rather dense network with high numbers of co-occurring taxa (Figure 4). Network complexity again substantially decreased at sites of lateral root primordia although by far not as much as at root tips, and finally increased again significantly in the lateral root zone with 16% lower modularity but 1.38, 2.26, and 1.65 fold increased numbers of nodes, links and average connectivity (avgK) compared to root primordia, respectively (Table 1). Positive co-occurrences by far dominated in all networks (Figure 4), but from bulk soil to lateral root primordia the percentage of negative correlations gradually decreased and only increased again to bulk soil level at sites with fully developed lateral roots. Most nodes were represented by bacteria, while Cercozoa and archaea made up a much smaller share of total nodes (Figures 4A–E). Archaea formed clear sub-network clusters in bulk soil and on lateral roots (Figures 4A,E).

**TABLE 1:** Topological features of empirical networks of bulk soil, root tip, root hair zone, lateral root primordia and lateral roots, including inter inter- and intra-domain and intra domain co-occurrences of bacteria, archaea, and Cercozoa.

	Empirical networks							
	
	Similarity threshold	Total number of nodes	Total number of links	*R*^2^ of power-law	Average degree (avgK)	Average clustering coefficient (avgCC)^b^	Average path distance (APD)^b^	Modularity (MOD)^b^
Bulk soil	0.76	172	597	0.954	6.942	0.411^a^	3.246^a^	0.553^a^
Root tip	0.76	211	279	0.994	2.645	0.181^a^	7.146^a^	0.787^a^
Root hair	0.76	511	6396	0.970	25.033	0.473^a^	2.914^a^	0.280^a^
Primordia	0.76	218	674	0.978	6.183	0.348^a^	3.643^a^	0.511^a^
Lateral	0.76	296	1521	0.988	10.277	0.405^a^	3.376^a^	0.431^a^

*The “*R*^2^ of power law” describes the proportion of variance explained under the assumption that the number of connections per node follow a power law function; the “average degree” is the average number of connections per node in the respective network. ^*a*^Significant difference (*p* < 0.001) in avgCC, APD, and MOD of empirical networks compared to random networks (Table 3), based on one sample Student’s *t* test. ^*b*^Significant difference (*p* < 0.001) in avgCC, APD and MOD between bulk soil, root tip, root hair zone, lateral root primordial, and lateral root networks using Student’s *t* test.*

**TABLE 2:** Means of topological features of 100 networks, generated by randomly rewiring nodes and links of empirical bulk soil, root tip, root hair zone, lateral root primordial, and lateral root networks.

Random networks			

	Average clustering coefficient (avgCC)	Average path distance (APD)	Modularity (MOD)
Bulk soil	0.14 ± 0.014	2.883 ± 0.045	0.314 ± 0.005
Root tip	0.017 ± 0.006	4.455 ± 0.113	0.657 ± 0.007
Root hair	0.321 ± 0.008	2.531 ± 0.013	0.133 ± 0.003
Primordia	0.132 ± 0.013	3.039 ± 0.043	0.334 ± 0.005
Lateral	0.202 ± 0.011	2.78 ± 0.029	0.234 ± 0.005

**FIGURE 5:**
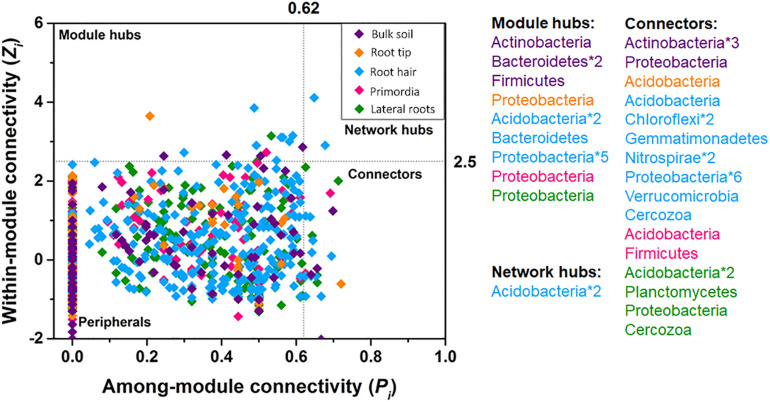
Within-module connectivity (*Z*_i_) and among-module connectivity (*P*_i_) of network nodes. Points exceeding thresholds of *Z*_i_ = 2.5 and/or *P*_i_ = 0.62 were identified as module hubs, network hubs or connectors.

Bacterial taxa exclusively acted as module hubs and network hubs in all five networks (Figure 5). Module hubs were represented by Actinobacteria, Bacteroidetes, and Firmicutes in bulk soil and Proteobacteria, Bacteriodetes, and Acidobacteria in rhizosphere networks. Network hubs were exclusively represented by Acidobacteria in the root hair zone (Figure 5). Cercozoan protists acted as connectors in the more complex community networks of root hair zone and lateral roots, together with a changing variety of bacterial taxa in different root regions (Figure 5).

**FIGURE 6:**
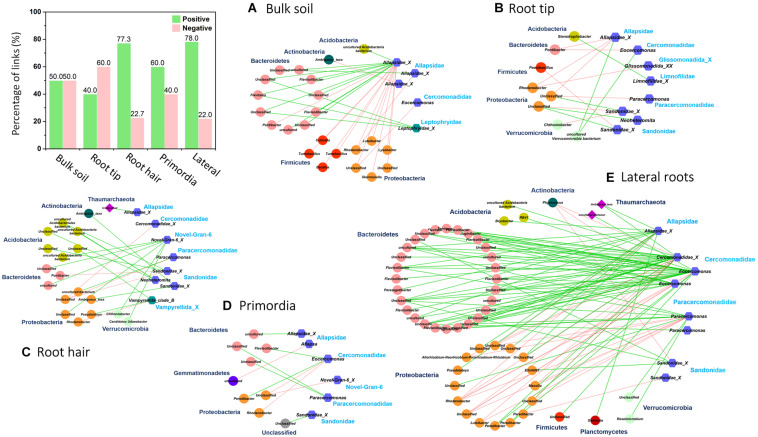
Bipartite microbial co-occurrence networks based on correlation analysis of bacteria (circles), archaea (rhombuses), and Cercozoa (hexagons) in **(A)** bulk soil and four different root regions of increasing age with **(B)** root tip, **(C)** root hair zone, **(D)** lateral root primordial, and **(E)** lateral roots. Node colors were mapped to the phylum level. Only positive (green edges) or negative (red edges) correlations of Cercozoa with bacteria or archaea were displayed. Nodes were clustered on family level based on their current taxonomy. The bar plot shows the percentage of positive and negative correlations between Cercozoa and bacteria/archaea in bulk soil and at the different root regions.

In order to identify potential trophic relationships between protists and prokaryotes, bipartite inter-domain associations between Cercozoa and prokaryotes were extracted from full networks along roots (Figure 6 and Table 3). The bulk soil bipartite “trophic network” revealed distinct phylogenetic patterns with positive co-occurrences of protists in Allapsidae and Leptophryidae with bacterial members of Bacteriodetes and negative correlations with Proteobacteria and Firmicutes (Figure 6). Although bipartite networks of root tips, the root hair zone and lateral root primordia always identified the same protistan taxa as potential predators, these networks appeared scattered and did not reveal distinct, repeatable patterns of co-occurrence with prokaryotes.

**TABLE 3:** Topological features of bipartite networks, only considering co-occurrences of protists with prokaryotes of bulk soil, root tip, root hair zone, lateral root primordial, and lateral roots.

	Number of protist OTUs	Number of bacterial/archaeal OTUs	Total number of nodes	Total number of associations	Connectance	Links per OTU
Bulk soil	5	23	28	28	0.243	1.000
Root tip	8	8	16	15	0.234	0.938
Root hair	8	19	27	22	0.145	0.815
Primordia	6	9	15	10	0.185	0.667
Lateral	8	54	62	109	0.252	1.758

*Connectance is the proportion of possible links that are established.*

In contrast, the highest complexity of bipartite networks was found on lateral roots, as in bulk soil showing again clearly positive co-occurrences of protists in Allapsidae, Cercomonadidae, and Paracercomonadidae with Bacteriodetes and negative correlations with Proteobacteria (Figure 6E). The percentage of negative correlations in bipartite trophic networks was always quite high, indicating potential predation effects, except in the root hair zone and lateral root region (Figure 6, bar chart). High numbers of prokaryotic OTUs, connectance, and links per species in bulk soil, declined at the root tip, remained low in the root hair zone and sites of lateral root primordia, but significantly increased at lateral roots (Table 3) indicating a decrease in network complexity from bulk soil to young root regions, followed by a large increase in network complexity at lateral roots.

## Discussion

The concept of a “core” microbiome implies highly deterministic processes in the assembly of rhizosphere communities and predictability of their plant-associated traits (Friesen et al., 2011; Oyserman et al., 2018). Recent studies have revealed much about the deterministic nature of species-specific rhizosphere microbiomes and their variation due to differences in soil type, plant developmental stage or plant genotype (Bouffaud et al., 2012; Peiffer et al., 2013; Chaparro et al., 2014; Bulgarelli et al., 2015; Edwards et al., 2015; Perez-Jaramillo et al., 2016, 2017; Rossmann et al., 2020), but much less is known on where and when a rhizosphere microbiome is formed along the root axis from a random bulk soil community (Di Battista-Leboeuf et al., 2003).

It has been assumed that microbiome members are already selected at root tips through mucilage and root border cells (Hawes et al., 2012; Baetz and Martinoia, 2014), while others emphasized the role of root exudates released at more distal regions along the root axis (Oger et al., 2004; Steinkellner et al., 2007; Hartmann et al., 2009; Li et al., 2016), or proposed that the plant microbiome assembles along the way from rhizosphere to endosphere, with the plant immune system at the rhizoplane playing a dominant role for community selection (Lundberg et al., 2012; Edwards et al., 2015).

Community assembly of the maize microbiome along the root axis showed more complex patterns than anticipated. Apart from OTU richness, which strongly decreased at root tips and slowly recovered toward older root regions, there was no gradual transitions of community composition along the longitudinal root axis. In order to understand the mechanisms of microbiome assembly, patterns of microbial alpha and beta diversity, as well as changes in the overall and trophic network structure, had to be equally taken into account.

At root tips, a decrease of OTU richness and alpha diversity of bacteria as compared to bulk soil has been repeatedly reported (Bulgarelli et al., 2012; Shi et al., 2015; Fan et al., 2017). The associated changes in species composition on maize root tips were confirmed by a clear shift in beta diversity compared to bulk soil (Figure 3A), and most likely can be attributed to the dominance of fast-growing copiotrophic users of energy-rich rhizodeposits (Schönwitz and Ziegler, 1989; Andrews and Harris, 2000; Benizri et al., 2007; Fierer et al., 2007). Additionally, there is growing evidence that root border cells and secreted mucilage at tips actively bind, immobilize and aggregate specific microbial cells before they become members of the microbiome (Humphris et al., 2005; Hawes et al., 2012; Baetz and Martinoia, 2014). Root tip communities, however, were characterized by the largest variation in OTU richness and beta diversity (Figures 2, 3B). This pattern suggests that different species gain dominance on individual root tips, demonstrating a substantial randomness in the outcome of early rhizosphere community assembly. These results are further supported by high evenness of prokaryote communities on root tips (Figure 2), indicative of a reduced dominance of specific taxa. Taken together, community structure had markedly changed from bulk soil to root tips, but rhizosphere microbiome composition was far from uniform at the early stages of community assembly.

Maize root exudation contributes 10–100 times more carbon to the rhizosphere than border cells and mucilage at root tips (Nguyen, 2003). Piñeros et al. (2002) reported a maximum release of organic acids by maize exudates about 5 cm beyond maize root tips. Thus, microorganisms in the root hair zone profit from maximum energy supply of rhizodeposition. Because root exudates contain a variety of plant species-specific metabolites and signal compounds with important functional roles in plant defense and symbiosis (Baetz and Martinoia, 2014) they have been suggested as a main driver for the selection of root-specific microorganisms (Badri and Vivanco, 2009; Hartmann et al., 2009; van Dam and Bouwmeester, 2016). However, Dini-Andreote et al. (2015) hypothesized that microbial dispersal in systems with abundant resource supply will lead to the dominance of neutral, stochastic assembly processes. A marked shift of beta diversity in the root hair zone compared with root tips reflects these rhizosphere-driven changes in community structure (Figure 3A). Nevertheless, maximum variation of OTU richness, alpha diversity, evenness (Figure 2), and high variation of beta diversity (Figure 3A) still show significant randomness in community assembly. An explanation is given by the root hair network structure (Figure 4C). Its low modularity with highest number of nodes and links reflects a large network with massive co-occurrences of taxa (Table 1), indicating a parallel unconstrained growth of large numbers of different microorganisms fueled by the high rhizodeposition (Figure 4C). Accordingly, selective forces through competition and predation will be at a minimum, and this is reflected in the bipartite trophic network of root hairs (Figure 6C). Although the bipartite trophic network indicates the co-occurrences of common protistan taxa with specific bacteria, its network structure was rather poor. It showed the lowest connectance of all root region networks, and the ratio of empirical network links to potential trophic links in the bipartite network increased more than 10-fold compared to bulk soil and root tips, demonstrating that predators were loosely linked to the overall network structure. Protistan beta diversity showed highest variation in the root hair zone (Figure 3B), indicating high variability in the dominance structure of predator communities among replicates. Thus despite the extraordinarily high co-occurrence of prokaryotes in the root hair zone, correlations of prokaryotes to protistan predators were sparse (Figure 6C and Table 3), suggesting that the predators followed an opportunistic feeding strategy and most likely exerted no top-down control over the exponential growth of bacteria. These assumptions are further supported by the high percentage of positive co-occurrences between protists and their potential prey in these networks (Figure 6, bar chart), suggesting that the key predators in bipartite networks mainly benefited from the abundant food supply. In agreement with the high resource-high stochasticity hypothesis of Dini-Andreote et al. (2015), our results indicate that areas of high rhizodeposition rather indiscriminately stimulated microbial growth and reproduction. This phenomenon is reflected in the high average degree and number of links between protists and prokaryotes in the lateral root network compared to networks of younger root regions.

A further microbial hotspot along the root axis are sites of lateral root emergence. In maize, the endodermis of the parent root gives rise to the epidermis of the lateral root (Lloret and Casero, 2002). Enzymes, defense compounds and other secondary metabolites are released at these sites by the breakage of lateral root primordia as earliest lateral roots through the outer root cell cortex (Ashford and McCully, 1973; Van Egeraat, 1975; Lloret and Casero, 2002) and were shown to attract a specific suite of microbial consumers (Jaeger et al., 1999; Baudoin et al., 2002; Park et al., 2004). Beta diversity of prokaryotes in the zone of lateral root emergence showed much less variability than in the root hair region (Figure 3), and their low evenness reflects a competitive community shift toward dominance of few taxa, both indicating the onset of deterministic processes of community assembly as energy supply from the root hair region had ceased. The much lower number of co-occurring taxa of the empirical network also points to a reduced availability of resources compared to the root hair region. The trophic network showed few correlations to potential prey bacteria, still indicating the absence of top-down control. While the quantity and composition of root metabolites has a direct influence on prokaryote communities, the latter in turn is expected to feed back on the community structure of their cercozoan consumers (Matz and Kjelleberg, 2005; Jousset, 2012; Song et al., 2015). The smaller ordination space in NMDS covered by prokaryotes in bulk soil and in older root sections as compared to protists likely reflects these indirect relationships (Figure 3B). The first-order laterals of maize are mostly short and reach their final length within 2–3 days (Fusseder, 1987; Pagès and Pellerin, 1994; Hochholdinger, 2009). Rhizodeposition occurs only at the distal root tips, because the fully developed root endodermis and exodermis prevent losses of cell compounds in this region (Neumann and Römheld, 2012). The scarcity of resource supply from rhizodeposition likely continues to favor deterministic processes of community assembly. Evidence comes from variation of beta diversity, which was lowest of all root regions, and the community structure showed no overlap with bulk soil communities (Figure 3A), demonstrating that a consistent root microbiome finally assembled. Co-occurrence network complexity considerably increased compared to root primordia, and the number of negative co-occurrences also rose, potentially indicating competitive exclusion. The bipartite trophic network between prokaryotes and protists on lateral roots showed clear phylogenetic patterns, as would be expected if different prokaryote taxa evolved different defensive traits that are phylogenetically clustered (Matz and Kjelleberg, 2005; Jousset, 2012; Song et al., 2015). Targeted experiments on protist-bacteria interactions demonstrate strong selection effects favoring grazing-resistant taxa (Koh et al., 2012), while reducing the dominance of fast-growing, less-defended competitors (Jousset et al., 2008).

Due to the increasingly applied role of rhizosphere microbiomes for enhancing plant productivity, a deeper mechanistic understanding of microbial assembly processes appears crucial. Community assembly in the rhizosphere is often solely viewed from the plant perspective as an increasingly plant-driven selection of microbial taxa from bulk soil to rhizosphere and rhizoplane. Our study emphasizes the importance of microbial interactions and the dynamic nature of the processes related to microbiome assembly along the root axis. Already the microbial communities on root tips differed significantly from bulk soil, but high variation in beta diversity showed substantial randomness in community structure between individual root tips. Contrary to our expectations, randomness persisted in the root hair zone, where community networks indicate rather indiscriminate growth of most taxa due to abundant resource supply. On the contrary, depletion of resources reduced variation within rhizosphere microbiomes and favored more structured network topologies, suggesting important roles of competition and predation in rhizosphere microbiome assembly.

## Data Availability Statement

The data have been submitted to the European Nucleotide Archive and are available under the accession number PRJEB40690.

## Author Contributions

MB designed the study. DV did the experimental framework. LR conducted the experiment, prepared the samples for sequencing, and performed the bioinformatics with the help of KD and data analysis assisted by KF (network analysis). BS, YC, and RS provided sequences of prokaryota. MB and LR wrote the manuscript with assistance of MW. PY and FH contributed significantly with their expertise on maize root development and exudation. All authors read and approved the manuscript.

## Conflict of Interest

The authors declare that the research was conducted in the absence of any commercial or financial relationships that could be construed as a potential conflict of interest.

## Footnotes

1 http://ieg4.rccc.ou.edu/mena/

2 http://mem.rcees.ac.cn:8081
