# Supplementary material for: Assembly Patterns of the Rhizosphere Microbiome Along the Longitudinal Root Axis of Maize (Zea mays L.)
Source: Front Microbiol. 2021 Feb 12;12:614501. doi: 10.3389/fmicb.2021.614501 (PMC7906986; doi:10.3389/fmicb.2021.614501)
Supplement: Supplementary file 1 [file Data_Sheet_1.docx]

# Supplement


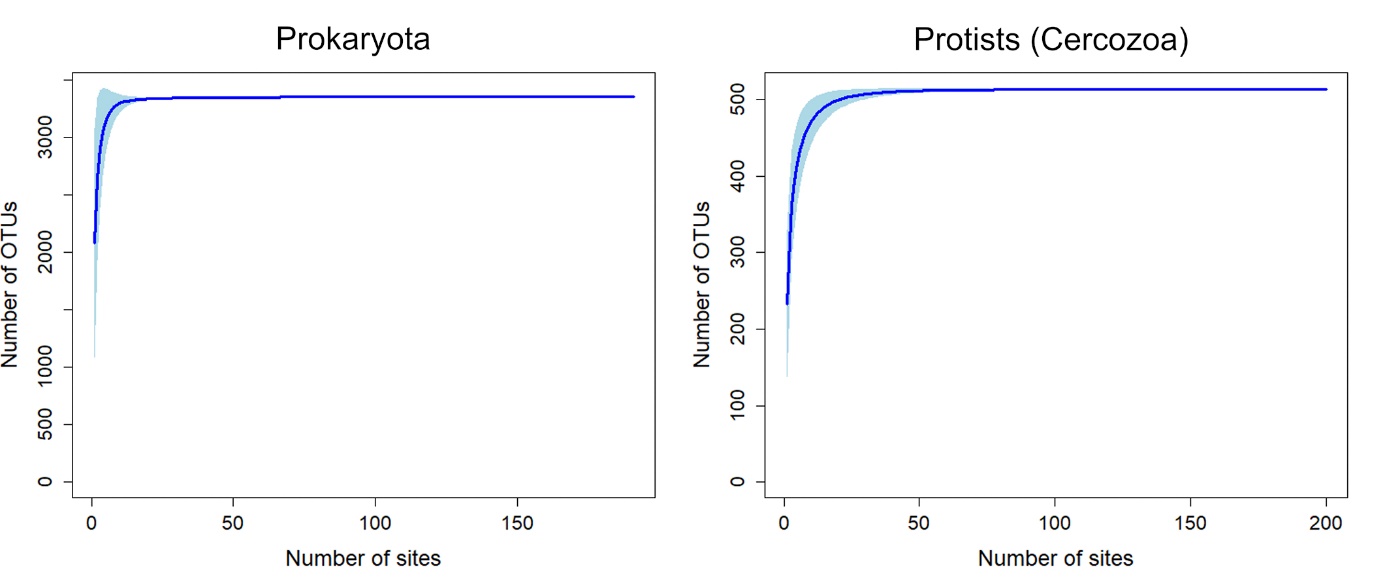
 **Spplementary Figure 1.** Accumulation curves describing the observed number of prokaryotic (left) and protistan (right) OTUs as a function of the sampling effort, saturation was reached.

**Supplementary Table 1.** Percent Coefficient of Variation (%CV) of OTU richness, Shannon entropy and Pielou evenness, calculated for bacteria/archaea and Cercozoa in bulk soil and at the four different root regions sampled.

|  | Bacteria/ Archaea | | | Cercozoa | | |
| --- | --- | --- | --- | --- | --- | --- |
|  | %CV of OTU richness | %CV of Shannon entropy | %CV of Pielou evenness | %CV of OTU richness | %CV of Shannon entropy | %CV of Pielou evenness |
| Bulk soil | 11.312 | 1.831 | 2.209 | 13.325 | 11.840 | 11.072 |
| Root tip | 30.865 | 10.677 | 7.895 | 21.040 | 19.343 | 17.344 |
| Root hair | 32.098 | 17.817 | 13.942 | 24.785 | 25.172 | 22.280 |
| Primordia | 21.341 | 10.594 | 8.283 | 19.322 | 14.379 | 13.038 |
| Lateral | 12.770 | 10.060 | 8.999 | 18.179 | 14.928 | 13.524 |

**Supplementary Table 2.** Adjusted p-values of pairwise comparison indicating differences in prokaryote community composition between samples from bulk soil, root tip, root hair zone, lateral root primordia and lateral roots.

| **Adjusted p-value** | **Root tip** | **Root hair** | **Primordia** | **Lateral** |
| --- | --- | --- | --- | --- |
| **Bulk soil** | 0.01* | 0.01* | 0.01* | 0.01* |
| **Root tip** |  | 0.01* | 0.01* | 0.01* |
| **Root hair** |  |  | 1 | 0.09 |
| **Primordia** |  |  |  | 0.03* |
| *Significant difference based on pairwise comparison of Bray-Curtis dissimilarities | | | | |

**Supplementary Table 3.** Adjusted p-values of pairwise comparison indicating differences in protist community composition between samples from bulk soil, root tip, root hair zone, lateral root primordia and lateral roots.

| **Adjusted p-value** | **Root tip** | **Root hair** | **Primordia** | **Lateral** |
| --- | --- | --- | --- | --- |
| **Bulk soil** | 0.1 | 0.04* | 0.01* | 0.01* |
| **Root tip** |  | 1 | 0.02* | 0.02* |
| **Root hair** |  |  | 0.74 | 0.01* |
| **Primordia** |  |  |  | 0.06 |
| *Significant difference based on pairwise comparison of Bray-Curtis dissimilarities. | | | | |
